# Quintic non-polynomial spline for time-fractional nonlinear Schrödinger equation

**DOI:** 10.1186/s13662-020-03021-0

**Published:** 2020-10-16

**Authors:** Qinxu Ding, Patricia J. Y. Wong

**Affiliations:** School of Electrical and Electronic Engineering, Nanyang Technological University, 50 Nanyang Avenue, Singapore, 639798 Singapore

**Keywords:** 65M12, 65N12, Quintic non-polynomial spline, Time-fractional derivative, Nonlinear Schrödinger equation

## Abstract

In this paper, we shall solve a time-fractional nonlinear Schrödinger equation by using the quintic non-polynomial spline and the *L*1 formula. The unconditional stability, unique solvability and convergence of our numerical scheme are proved by the Fourier method. It is shown that our method is sixth order accurate in the spatial dimension and $(2-\gamma )$th order accurate in the temporal dimension, where *γ* is the fractional order. The efficiency of the proposed numerical scheme is further illustrated by numerical experiments, meanwhile the simulation results indicate better performance over previous work in the literature.

## Introduction

In the past few decades, fractional differential equations have gained much importance due to their usefulness in modeling phenomena in various areas such as physics, engineering, finance, biology and chemistry [12, 33, 46, 51]. To cite some recent developments: in 2019 Jajarmi and Baleanu [26] studied a general form of fractional optimal control problems involving fractional derivative with singular or non-singular kernel; Jothimani et al. [30] discussed an exact controllability of nondensely defined nonlinear fractional integrodifferential equations with the Hille–Yosida operator; Valliammal et al. [61] studied the existence of mild solutions of fractional-order neutral differential system with state-dependent delay in Banach space. In 2020 Jajarmi et al. [28] investigated a fractional version of SIRS model for the HRSV disease; Baleanu et al. [2] proposed a new fractional model for the human liver involving the Caputo–Fabrizio fractional derivative; Baleanu et al. [3] studied the fractional features of a harmonic oscillator with position-dependent mass; Sajjadi et al. [54] discussed the chaos control and synchronization of a hyperchaotic model in both the frameworks of classical and of fractional calculus; Jajarmi and Baleanu [27] proposed a new iterative method to generate the approximate solution of nonlinear fractional boundary value problems in the form of uniformly convergent series; Shiri et al. [56] employed discretized collocation methods for a class of tempered fractional differential equations with terminal value problems; Tuan et al. [60] tackled the problem of finding the solution of a multi-dimensional time-fractional reaction-diffusion equation with nonlinear source from the final value data; Li et al. [44] proposed a new approximation for the generalized Caputo fractional derivative based on WSGL formula and solved a generalized fractional sub-diffusion problem; Gao et al. [19] studied the epidemic predictability for the novel coronavirus (2019-nCoV) pandemic by analyzing a time-fractional model and finding its solution by a *q*-homotopy analysis transform method; Gao et al. [20] investigated the infection system of the novel coronavirus (2019-nCoV) with a nonlocal operator defined in the Caputo sense; Gao et al. [21] tackled the fractional Phi-four equation by using a *q*-homotopy analysis transform method numerically; Sabir et al. [53] presented a novel meta-heuristic computing solver for solving the singular three-point second-order boundary value problems using artificial neural networks.

The subject of the present work, the Schrödinger equation, was first proposed by the Austrian physicist Erwin Schrödinger in 1926 [55]. It is a fundamental equation in quantum physics that describes the evolution of the position-space wave function of a particle. In fact, the nonlinear Schrödinger equations describe a wide class of physical phenomena such as models of protein dynamics, self-focusing in laser pulses and nonlinear fiber optics [1, 11, 17, 58]. The Schrödinger equation has also been generalized to fractional differential equations. In 2000, Laskin [34] generalized the non-fractional Schrödinger equation to a space-fractional Schrödinger equation by using the Feynman path integrals over the Lévy trajectories and replacing the quantum Riesz derivative with the Laplace operator. Later, in 2004 Naber [50] proposed a different generalization by changing the first order time-derivative to a Caputo fractional derivative—this time-fractional Schrödinger equation has been used to describe fractional quantum mechanical behavior. In 2010, Muslih et al. [49] obtained a fractional Schrödinger equation by using a fractional variational principle and a fractional Klein–Gordon equation. In 2017, Gómez-Aguilar and Baleanu [23] presented an alternative model of fractional Schrödinger equation involving Caputo–Fabrizio fractional operator.

Many researchers pay attention to the numerical treatment of fractional Schrödinger equations. For the *space-fractional* Schrödinger equation: a linear implicit conservative difference scheme of order $O(\tau ^{2}+h^{2})$ has been proposed in [62] for the case of a coupled nonlinear Schrödinger equation, where *τ* is the temporal step size and *h* is the spatial step size; Zhao et al. [65] have used a compact operator to approximate the Riesz derivative, and the proposed linearized difference scheme for a two-dimensional nonlinear space-fractional Schrödinger equation can achieve $O(\tau ^{2}+\bar{h}^{4})$, where $\bar{h}=\max \{h_{1}, h_{2}\}$, $h_{1}$ and $h_{2}$ are the spatial step sizes in the *x* and *y* dimensions, respectively; Wang and Huang [64] have presented a conservative linearized difference scheme, which can achieve the order of $O(\tau ^{2}+h^{2})$; a collocation method has been applied to a multi-dimensional space-time variable-order fractional Schrödinger equation in [5]; a fourth-order implicit time-discretization scheme based on the exponential time differencing approach together with a fourth-order compact scheme in space have been proposed in [31], the method is of order $O(\tau ^{4}+h^{4})$; Li et al. [37] have used a fast linearized conservative finite element method to solve the coupled type equation; Wang and Xiao [63] have proposed an efficient conservative scheme for the fractional Klein–Gordon–Schrödinger equation with central difference and Crank–Nicolson scheme, their method can achieve $O(\tau ^{2}+h^{2})$. It is also noted that Hashemi and Akgül [24] have utilized Nucci’s reduction method and the simplest equation method to extract analytical solutions specially of soliton kinds of nonlinear Schrödinger equations in both time and space fractional terms.

For the *time-fractional* Schrödinger equation: Khan et al. [32] have applied the homotopy analysis method; Mohebbi et al. [47] have used the Kansa approach to approximate the spatial derivative and *L*1 discretization to approximate the Caputo time-fractional derivative; a Krylov projection method has been developed in [22]; a Jacobi spectral collocation method has been applied to a multi-dimensional time-fractional Schrödinger equation in [4]; a quadratic B-spline Galerkin method combined with *L*1 discretization scheme has been proposed in [16]; a linearized *L*1-Galerkin finite element method has been used in [35] for a multi-dimensional nonlinear time-fractional Schrödinger equation; a cubic non-polynomial spline method combined with *L*1 discretization has been proposed in [36] and the stability has been shown by the Fourier method, the convergence order is not proved but is observed from numerical experiments to be $O(\tau ^{2-\gamma }+h^{4})$.

Motivated by the above research, in this paper we consider the following time-fractional nonlinear Schrödinger equation:

1.1 $$ \textstyle\begin{cases}\displaystyle i\,{}^{C}_{0}{D}^{\gamma }_{t} u(x,t)+ \frac{\partial ^{2} u(x,t)}{\partial x^{2}}+\lambda \big\vert u(x,t) \big\vert ^{2} u(x,t)=f(x,t), \quad (x,t)\in [0,L]\times [0,T], \\ u(x,0)=A_{0}(x), \quad x\in [0,L], \\ u(0,t)=A_{1}(t), \qquad u(L,t)=A_{2}(t), \quad t\in [0,T], \end{cases} $$

where *λ* is a real constant, $f(x,t)$, $A_{0}(x)$, $A_{1}(t)$, $A_{2}(t)$ are continuous functions with $A_{0}(0)=A_{1}(0)$ and $A_{0}(L)=A_{2}(0)$, and ${}^{C}_{0}{D}^{\gamma }_{t} u(x,t)$ is the Caputo fractional derivative of order $\gamma \in (0,1)$ defined by [12, 51]

1.2 $$ {}^{C}_{0}{D}^{\gamma }_{t} u(x,t)= \frac{1}{\Gamma (1-\gamma )} \int _{0}^{t} (t-s)^{-\gamma } \frac{\partial u(x,s)}{\partial s}\,ds. $$

We shall employ a quintic non-polynomial spline together with *L*1 discretization to solve (1.1). The stability, unique solvability and convergence of our numerical scheme are then proved by the *Fourier method*—we note that this method of proof is *rare* for numerical methods of time/space-fractional Schrödinger equation, especially in establishing the convergence order; on the other hand, the energy method has been commonly used to show the convergence of numerical methods for space-fractional Schrödinger equation [31, 62–65]. By the Fourier method, it is shown that our method is of order $O(\tau ^{2-\gamma }+h^{6})$—this *improves* the *spatial convergence* achieved by other methods for time-fractional Schrödinger equation. Further, on the choices of our tools, we have observed in several different problems that a non-polynomial spline usually exhibits a better approximation than a polynomial spline because of its parameter [13, 15, 25, 38–43, 52, 57]; while *L*1 discretization is a stable and widely used approximation for the Caputo fractional derivative [15, 18, 29, 38, 48].

The organization of this paper is as follows. We derive the numerical scheme in Sect. 2. The stability, unique solvability and convergence are established by the Fourier method in Sects. 3, 4 and 5 respectively. In Sect. 6, we present three examples to verify the efficiency of our numerical scheme and to compare with other methods in the literature. Finally, a conclusion is drawn in Sect. 7.

## Derivation of the numerical scheme

In this section, we shall develop a numerical scheme for problem (1.1) by using quintic non-polynomial spline and *L*1 discretization. The details of quintic non-polynomial spline will be presented first.

Let

2.1 $$ \bar{P}: 0=x_{0}< x_{1}< \cdots < x_{M}=L \quad \mbox{and}\quad \bar{P}': 0=t_{0}< t_{1}< \cdots < t_{N}=T $$

be uniform meshes of the spatial interval $[0,L]$ with step size $h=\frac{L}{M}$ and the temporal interval $[0,T]$ with step size $\tau =\frac{T}{N}$, respectively. For any given function $y(x,t)$, we denote $y(x_{j},t_{n})$ by $y_{j}^{n}$, and $y(x_{j},t)$ by $y_{j}$ for fixed *t*.

Let $u(x,t)$ denote the exact solution of (1.1) and $U_{j}^{n}$ denote the numerical approximation of $u_{j}^{n}$. We shall set $U_{j}^{n}$ to be the value of the quintic non-polynomial spline at $(x_{j},t_{n})$. We define the quintic non-polynomial spline as follows.

### Definition 2.1

([57])

Let $t=t_{n}$, $1\leq n \leq N$ be fixed. For a given mesh *P̄*, we say $P_{n}(x)$ is the *quintic non-polynomial spline with parameter* $k~(>0)$ if $P_{n}(x) \in C^{(4)}[0,L]$, $P_{n}(x)$ has the form $\operatorname{span}\{1,x,x^{2},x^{3},\sin (kx), \cos (kx)\}$ and its restriction $P_{j,n}(x)$ on $[x_{j},x_{j+1}]$, $0\leq j \leq M-1$ satisfies

2.2 $$ \textstyle\begin{cases} P_{j,n}(x_{j})=U_{j}^{n}, \qquad P_{j,n}(x_{j+1})=U_{j+1}^{n}, \\ P^{(2)}_{j,n}(x_{j})=W_{j}^{n}, \qquad P^{(2)}_{j,n}(x_{j+1})=W_{j+1}^{n}, \\ P^{(4)}_{j,n}(x_{j})=F_{j}^{n}, \qquad P^{(4)}_{j,n}(x_{j+1})=F_{j+1}^{n}. \end{cases} $$

From the above definition, we can express $P_{j,n}(x)$ on $[x_{j},x_{j+1}]$, $0\leq j\leq M-1$ as

2.3 $$ P_{j,n}(x)=\overline{a}_{j}^{n}+ \overline{b}_{j}^{n}(x-x_{j})+ \overline{c}_{j}^{n}(x-x_{j})^{2}+ \overline{d}_{j}^{n}(x-x_{j})^{3}+ \overline{e}_{j}^{n}\sin k(x-x_{j})+ \overline{f}_{j}^{n}\cos k(x-x_{j}). $$

Denote $\omega =kh$. Using (2.2), a direct computation gives

2.4 $$ \begin{aligned} & \overline{a}_{j}^{n}=U_{j}^{n}- \frac{F_{j}^{n}}{k^{4}}, \\ & \overline{b}_{j}^{n}=\frac{U_{j+1}^{n}-U_{j}^{n}}{h}+ \frac{F_{j}^{n}-F_{j+1}^{n}}{\omega k^{3}}-\frac{h}{6} \bigl(2W_{j}^{n}+W_{j+1}^{n} \bigr)- \frac{h}{6k^{2}} \bigl(2F_{j}^{n}+F_{j+1}^{n} \bigr), \\ & \overline{c}_{j}^{n}=\frac{1}{2} \biggl(W_{j}^{n}+ \frac{F_{j}^{n}}{k^{2}} \biggr), \\ & \overline{d}_{j}^{n}=\frac{W_{j+1}^{n}-W_{j}^{n}}{6h}+ \frac{F_{j+1}^{n}-F_{j}^{n}}{6\omega k}, \\ & \overline{e}_{j}^{n}= \frac{F_{j+1}^{n}-F_{j}^{n}\cos \omega }{k^{4}\sin \omega }, \\ & \overline{f}_{j}^{n}=\frac{F_{j}^{n}}{k^{4}}. \end{aligned} $$

Using the continuity of the first and third derivatives of the spline at $x=x_{j+1}$, i.e., $P^{(1)}_{j,n}(x_{j+1})=P^{(1)}_{j+1,n}(x_{j+1})$ and $P^{(3)}_{j,n}(x_{j+1})=P^{(3)}_{j+1,n}(x_{j+1})$, we obtain the following relations for $1\leq j\leq M-1$:

2.5 $$ \begin{aligned} (\mathrm{a})&\quad W_{j-1}^{n}+4W_{j}^{n}+W_{j+1}^{n} \\ &\hphantom{(\mathrm{a})}\quad = \frac{6}{h^{2}} \bigl(U_{j-1}^{n}-2U_{j}^{n}+U_{j+1}^{n} \bigr)-6h^{2} \bigl(\alpha _{1}F_{j-1}^{n}+2 \beta _{1}F_{j}^{n}+\alpha _{1}F_{j+1}^{n} \bigr), \\ (\mathrm{b})&\quad W_{j-1}^{n}-2W_{j}^{n}+W_{j+1}^{n}=h^{2} \bigl( \alpha F_{j-1}^{n}+2\beta F_{j}^{n}+ \alpha F_{j+1}^{n} \bigr), \end{aligned} $$

where

2.6 $$ \begin{aligned} & \alpha =\frac{1}{\omega ^{2}} \biggl( \frac{\omega }{\sin \omega }-1 \biggr), \qquad {}\beta = \frac{1}{\omega ^{2}} \biggl(1- \frac{\omega \cos \omega }{\sin \omega } \biggr), \\ & \alpha _{1}=\frac{1}{6\omega ^{2}}+ \frac{1}{\omega ^{4}}- \frac{1}{\omega ^{3} \sin \omega }, \qquad {}\beta _{1}=\frac{1}{3\omega ^{2}}-1+ \frac{\cos \omega }{\omega ^{3} \sin \omega }. \end{aligned} $$

Note that the consistency relation for (2.5)(b) will lead to $2(\alpha +\beta )=1$. Manipulating (2.5)(a) and (2.5)(b), we can easily get

2.7 $$ \begin{aligned} &p W_{j+2}^{n}+q W_{j+1}^{n}+s W_{j}^{n}+q W_{j-1}^{n}+p W_{j-2}^{n} \\ &\quad = \frac{1}{h^{2}} \bigl[\alpha U_{j+2}^{n}+2( \beta - \alpha )U_{j+1}^{n}+2(\alpha -2\beta )U_{j}^{n}+2(\beta -\alpha )U_{j-1}^{n}+ \alpha U_{j-2}^{n} \bigr], \\ &\qquad 2\leq j \leq M-2, \end{aligned} $$

where

2.8 $$ p=\alpha _{1}+\frac{\alpha }{6},\qquad q=2 \biggl[ \frac{2\alpha +\beta }{6}-(\alpha _{1}-\beta _{1}) \biggr],\qquad s=2 \biggl[\frac{\alpha +4\beta }{6}+(\alpha _{1}-2\beta _{1}) \biggr]. $$

Using the quintic non-polynomial spline to approximate the exact solution $u(x,t)$ of (1.1), the spline relation (2.7) leads to

2.9 $$ \begin{aligned} & p \frac{\partial ^{2} u_{j+2}^{n}}{\partial x^{2}}+q \frac{\partial ^{2} u_{j+1}^{n}}{\partial x^{2}}+s \frac{\partial ^{2} u_{j}^{n}}{\partial x^{2}}+q \frac{\partial ^{2} u_{j-1}^{n}}{\partial x^{2}}+p \frac{\partial ^{2} u_{j-2}^{n}}{\partial x^{2}} \\ &\quad = \frac{1}{h^{2}} \bigl[\alpha u_{j+2}^{n}+2( \beta - \alpha )u_{j+1}^{n}+2(\alpha -2\beta )u_{j}^{n}+2(\beta -\alpha )u_{j-1}^{n}+ \alpha u_{j-2}^{n} \bigr]+\Upsilon _{j}^{n}, \\ &\qquad 2\leq j \leq M-2, \end{aligned} $$

where $\Upsilon _{j}^{n}$ is the local truncation error in the spatial dimension. The next lemma gives a result on this error.

### Lemma 2.1

*For any fixed* $t=t_{n}$, $1\leq n\leq N$, *let* $u(x,t_{n})\in C^{(8)}[0,L]$. *If*

2.10 $$ p=\frac{1}{12}\alpha -\frac{1}{240},\qquad q=\frac{2}{3} \alpha + \frac{1}{10},\qquad s=-\frac{3}{2}\alpha + \frac{97}{120}, $$

*then the local truncation error* $\Upsilon _{j}^{n}$ *associated with the spline relation* (2.9) *satisfies*

2.11 $$ \Upsilon _{j}^{n}=O \bigl(h^{6} \bigr),\quad 2 \leq j\leq M-2. $$

### Proof

We carry out the Taylor expansion at $x=x_{j}$ in (2.9), this gives

$$ \Upsilon _{j}^{n}=Z_{1} \frac{\partial ^{2} u_{j}^{n}}{\partial x^{2}}+Z_{2} h^{2} \frac{\partial ^{4} u_{j}^{n}}{\partial x^{4}}+Z_{3}h^{4} \frac{\partial ^{6} u_{j}^{n}}{\partial x^{6}}+O \bigl(h^{6} \bigr), $$

where

$$\begin{aligned}& Z_{1}=2(\alpha +\beta )-2p-2q-s,\qquad Z_{2}= \frac{1}{6}(7\alpha + \beta )-4p-q, \\& Z_{3}= \frac{1}{180}(31\alpha +\beta )-\frac{4}{3}p- \frac{1}{12}q. \end{aligned}$$

To achieve the highest order of $O(h^{6})$, we set $Z_{1}=Z_{2}=Z_{3}=0$, which together with the consistency relation $2(\alpha +\beta )=1$, gives (2.10) and (2.11). □

A similar result to Lemma 2.1 has also been obtained in [38].

### Remark 2.1

In order to compute the numerical solution $U_{j}^{n}$, $1\leq j\leq M-1$, we need another two equations besides (2.7) or (2.9). We consider the following equations which incorporate the boundary conditions in (1.1):

2.12 $$ \begin{aligned} (\mathrm{a})&\quad b_{0} \frac{\partial ^{2} u_{0}^{n}}{\partial x^{2}}+b_{1} \frac{\partial ^{2} u_{1}^{n}}{\partial x^{2}}+b_{2} \frac{\partial ^{2} u_{2}^{n}}{\partial x^{2}}+b_{3} \frac{\partial ^{2} u_{3}^{n}}{\partial x^{2}}+b_{4} \frac{\partial ^{2} u_{4}^{n}}{\partial x^{2}} \\ &\hphantom{(\mathrm{a})} \quad =\frac{1}{h^{2}} \bigl(a_{0}u_{0}^{n}+a_{1}u_{1}^{n}+a_{2}u_{2}^{n}+a_{3}u_{3}^{n}+a_{4}u_{4}^{n} \bigr)+ \Upsilon _{1}^{n}, \\ (\mathrm{b})&\quad b_{M-4} \frac{\partial ^{2} u_{M-4}^{n}}{\partial x^{2}}+b_{M-3} \frac{\partial ^{2} u_{M-3}^{n}}{\partial x^{2}}+b_{M-2} \frac{\partial ^{2} u_{M-2}^{n}}{\partial x^{2}}+b_{M-1} \frac{\partial ^{2} u_{M-1}^{n}}{\partial x^{2}}+b_{M} \frac{\partial ^{2} u_{M}^{n}}{\partial x^{2}} \\ &\hphantom{(\mathrm{b})}\quad =\frac{1}{h^{2}} \bigl(a_{M-4}u_{M-4}^{n}+a_{M-3}u_{M-3}^{n}+a_{M-2}u_{M-2}^{n}+a_{M-1}u_{M-1}^{n}+a_{M}u_{M}^{n} \bigr) \\ &\hphantom{(\mathrm{b})}\qquad{}+ \Upsilon _{M-1}^{n}, \end{aligned} $$

where $\Upsilon _{1}^{n}$ and $\Upsilon _{M-1}^{n}$ are local truncation errors in the spatial dimension, and the constant $a_{i}$ and $b_{i}$ have to be computed such that

2.13 $$ \Upsilon _{j}^{n}=O \bigl(h^{6} \bigr),\quad j=1, M-1. $$

By carrying out Taylor expansion at $x=x_{2}$ and $x=x_{M-2}$ in (2.12)(a) and (2.12)(b), respectively, we get the following which satisfies (2.13):

2.14 $$\begin{aligned}& (a_{0},a_{1},a_{2},a_{3},a_{4})=(a_{M},a_{M-1},a_{M-2},a_{M-3},a_{M-4})= \biggl(\frac{1}{10},\frac{3}{5},-\frac{7}{5}, \frac{3}{5},\frac{1}{10} \biggr), \end{aligned}$$

2.15 $$\begin{aligned}& (b_{0},b_{1},b_{2},b_{3},b_{4})=(b_{M},b_{M-1},b_{M-2},b_{M-3},b_{M-4})= \biggl(\frac{1}{240},\frac{1}{6},\frac{79}{120}, \frac{1}{6}, \frac{1}{240} \biggr). \end{aligned}$$

### Remark 2.2

If we let the spline parameter $\alpha =\frac{1}{10}$ in (2.9), then $\text{(2.9)}|_{j=2}$ and $\text{(2.9)}|_{j=M-2}$ are simply the same as (2.12)(a) and (2.12)(b), respectively. Therefore, we should have $\alpha \neq \frac{1}{10}$.

To simplify the notations of the spline relations (2.9) and (2.12), we introduce the following definition.

### Definition 2.2

For $y=(y_{1},\ldots ,y_{M-1})$, we define the operators ∧ and ∧_1_ by

$$ \wedge y_{j}= \textstyle\begin{cases} b_{0}y_{0}+b_{1}y_{1}+b_{2}y_{2}+b_{3}y_{3}+b_{4}y_{4} , &j=1, \\ py_{j-2}+qy_{j-1}+sy_{j}+qy_{j+1}+py_{j+2}, & 2\leq j\leq M-2, \\ b_{M-4}y_{M-4}+b_{M-3}y_{M-3}+b_{M-2}y_{M-2}+b_{M-1}y_{M-1}+b_{M}y_{M} , & j=M-1, \end{cases} $$

and

$$ \wedge _{1} y_{j}= \textstyle\begin{cases} \displaystyle \frac{1}{h^{2}}(a_{0}y_{0}+a_{1}y_{1}+a_{2}y_{2}+a_{3}y_{3}+a_{4}y_{4}), &j=1, \\ \displaystyle \frac{1}{h^{2}}\bigl[\alpha y_{j-2}+2(\beta -\alpha )y_{j-1}+2( \alpha -2\beta )y_{j}+2(\beta -\alpha )y_{j+1}+\alpha y_{j+2}\bigr], & 2 \leq j\leq M-2, \\ \displaystyle \frac{1}{h^{2}}(a_{M-4}y_{M-4}+a_{M-3}y_{M-3}+a_{M-2}y_{M-2}+a_{M-1}y_{M-1}+a_{M}y_{M}), & j=M-1. \end{cases} $$

### Remark 2.3

In view of Definition 2.2, (2.11) and (2.13), the spline relations (2.9) and (2.12) can be presented as

2.16 $$ \wedge \frac{\partial ^{2} u_{j}^{n}}{\partial x^{2}}=\wedge _{1}u_{j}^{n}+O \bigl(h^{6} \bigr), \quad 1\leq j\leq M-1. $$

The next lemma gives the *L*1 discretization for the Caputo fractional derivative.

### Lemma 2.2

([18, 59])

*Let* $0<\gamma <1$ *and* $x=x_{j}$ *be fixed*. *If* $u(x_{j},t)\in C^{(2)}[0,t_{n}]$, *then we have*

2.17 $$ {}^{C}_{0}{D}_{t}^{\gamma } u(x_{j},t_{n})=\mu \sum_{k=0}^{n-1} R_{n,k}^{ \gamma } \bigl(u_{j}^{k+1}-u_{j}^{k} \bigr) +O \bigl(\tau ^{2-\gamma } \bigr), $$

*where* *τ* *is the temporal step size*,

2.18 $$ \mu =\frac{1}{\tau ^{\gamma }\Gamma (2-\gamma )},\qquad R_{n,k}^{ \gamma }=(n-k)^{1-\gamma }-(n-k-1)^{1-\gamma },\quad 0\leq k \leq n-1. $$

### Lemma 2.3

([9, 18])

*For* $R_{n,k}^{\gamma }$ *defined in* (2.18), *we have*

2.19 $$ \begin{aligned} (\mathrm{a})&\quad 1=R_{n,n-1}^{\gamma }>R_{n,n-2}^{\gamma }> \cdots >R_{n,k}^{\gamma }> \cdots >R_{n,1}^{\gamma }>R_{n,0}^{\gamma }>0, \\ (\mathrm{b})&\quad R_{n,k}^{\gamma }>\frac{1-\gamma }{(n-k)^{\gamma }},\quad 0 \leq k \leq n-1. \end{aligned} $$

We are now ready to derive the numerical scheme for (1.1). To begin, we discretize (1.1) at $(x_{j},t_{n})$ to get

2.20 $$ i\,{}^{C}_{0}{D}^{\gamma }_{t} u(x_{j},t_{n})+ \frac{\partial ^{2} u(x_{j},t_{n})}{\partial x^{2}}+\lambda \bigl\vert u(x_{j},t_{n}) \bigr\vert ^{2} u(x_{j},t_{n})=f(x_{j},t_{n}). $$

Using the *L*1 discretization (2.17) in (2.20), we obtain

2.21 $$ \frac{\partial ^{2} u(x_{j},t_{n})}{\partial x^{2}}=-i\mu \sum_{k=0}^{n-1}R_{n,k}^{ \gamma } \bigl(u_{j}^{k+1}-u_{j}^{k} \bigr)-\lambda \bigl\vert u_{j}^{n} \bigr\vert ^{2}u_{j}^{n}+f_{j}^{n}+O \bigl( \tau ^{2-\gamma } \bigr). $$

Next, applying the operator ∧ to (2.21) yields

$$ \wedge \frac{\partial ^{2} u(x_{j},t_{n})}{\partial x^{2}}=-i\mu \sum_{k=0}^{n-1}R_{n,k}^{\gamma } \bigl(\wedge u_{j}^{k+1}-\wedge u_{j}^{k} \bigr)- \lambda \wedge \bigl( \bigl\vert u_{j}^{n} \bigr\vert ^{2}u_{j}^{n} \bigr)+\wedge f_{j}^{n}+O \bigl( \tau ^{2-\gamma } \bigr). $$

Noting (2.16), it follows that

$$ \wedge _{1} u_{j}^{n}=-i\mu \sum _{k=0}^{n-1}R_{n,k}^{\gamma } \bigl(\wedge u_{j}^{k+1}- \wedge u_{j}^{k} \bigr)-\lambda \wedge \bigl( \bigl\vert u_{j}^{n} \bigr\vert ^{2}u_{j}^{n} \bigr)+\wedge f_{j}^{n}+O \bigl(h^{6}+\tau ^{2-\gamma } \bigr). $$

Upon rearranging the above relation, we have

2.22 $$ \begin{aligned} &\wedge _{1} u_{j}^{n}+i\mu \wedge u_{j}^{n}+\lambda \wedge \bigl( \bigl\vert u_{j}^{n} \bigr\vert ^{2}u_{j}^{n} \bigr) \\ &\quad =i\mu \wedge u_{j}^{n-1}-i\mu \sum _{k=0}^{n-2}R_{n,k}^{ \gamma } \bigl(\wedge u_{j}^{k+1}-\wedge u_{j}^{k} \bigr)+\wedge f_{j}^{n}+O \bigl(h^{6}+ \tau ^{2-\gamma } \bigr). \end{aligned} $$

After omitting the error and replacing the exact solution *u* with the numerical solution *U*, we get the numerical scheme

2.23 $$ \begin{aligned} &\wedge _{1} U_{j}^{n}+i \mu \wedge U_{j}^{n}+\lambda \wedge \bigl( \bigl\vert U_{j}^{n} \bigr\vert ^{2}U_{j}^{n} \bigr) \\ &\quad =i\mu \wedge U_{j}^{n-1}-i\mu \sum _{k=0}^{n-2}R_{n,k}^{ \gamma } \bigl(\wedge U_{j}^{k+1}-\wedge U_{j}^{k} \bigr)+\wedge f_{j}^{n}, \quad 1\leq j\leq M-1, 1\leq n\leq N, \end{aligned} $$

with

2.24 $$ \textstyle\begin{cases} U_{j}^{0}=A_{0}(x_{j}), \quad 0\leq j\leq M, \\ U_{0}^{n}=A_{1}(t_{n}), \qquad U_{M}^{n}=A_{2}(t_{n}), \quad 1\leq n \leq N. \end{cases} $$

### Remark 2.4

It is obvious that our scheme (2.23) is a nonlinear scheme. To linearize (2.23), following [45] we shall use the iterative algorithm

2.25 $$ \begin{aligned} &{}\wedge _{1} U_{j}^{n(r+1)}+i \mu \wedge U_{j}^{n(r+1)}+\lambda \wedge \bigl( \bigl\vert U_{j}^{n(r)} \bigr\vert ^{2}U_{j}^{n(r+1)} \bigr) \\ &\quad =i\mu \wedge U_{j}^{n-1}-i\mu \sum _{k=0}^{n-2}R_{n,k}^{\gamma } \bigl( \wedge U_{j}^{k+1}-\wedge U_{j}^{k} \bigr)+\wedge f_{j}^{n}, \quad 1\leq j \leq M-1, 1\leq n\leq N, \end{aligned} $$

where $U_{j}^{n(r)}$ is the *r*th iterate of $U_{j}^{n}$, and

2.26 $$ U_{j}^{n(0)}= \textstyle\begin{cases} U_{j}^{n-1}, &n=1, \\ 2U_{j}^{n-1}-U_{j}^{n-2}, & 2\leq n\leq N. \end{cases} $$

Note that the scheme (2.25) is linear in $U^{n(r+1)}$. Hence, instead of (2.23)–(2.24), in practice we shall employ the linearized iterative scheme (2.25) with

2.27 $$ \textstyle\begin{cases} U_{j}^{0(r+1)}=A_{0}(x_{j}), \quad 0\leq j\leq M, \\ U_{0}^{n(r+1)}=A_{1}(t_{n}),\qquad U_{M}^{n(r+1)}=A_{2}(t_{n}),\quad 1\leq n\leq N. \end{cases} $$

For practical purposes, we would certainly need a stopping criterion to get $U_{j}^{n(r+1)}$ to a desired accuracy. An example of such a stopping criterion is

2.28 $$ \frac{ \vert U_{j}^{n(r+1)}-U_{j}^{n(r)} \vert }{ \vert U_{j}^{n(r)} \vert }\leq \mbox{a small constant}. $$

In fact, we shall use the above stopping criterion with the small constant as $1\times 10^{-6}$ for our numerical simulations in Sect. 6.

## Stability analysis of the numerical scheme

In this section, we shall analyze the stability of the scheme (2.23)–(2.24) via the Fourier method [8, 10]. Noting from Remark 2.4 that in practice we employ the linearized scheme (2.25)–(2.27) instead, effectively this means that in (2.23) we linearize the nonlinear term $|U|^{2}U$ by replacing $|U|^{2}$ with a local constant *κ*. Note that a similar linearization technique has also been used in [14, 15]. With the linearization, we can rewrite (2.23)–(2.24) as

3.1 $$ \textstyle\begin{cases} \wedge _{1} U_{j}^{n}+i\mu \wedge U_{j}^{n}+\lambda \kappa \wedge U_{j}^{n} \\ \quad =i \mu \wedge U_{j}^{n-1}-i\mu \sum_{k=0}^{n-2}R_{n,k}^{\gamma }(\wedge U_{j}^{k+1}- \wedge U_{j}^{k})+\wedge f_{j}^{n},\quad 1\leq j\leq M-1, \\ U_{j}^{0}=A_{0}(x_{j}),\quad 0\leq j\leq M, \\ U_{0}^{n}=A_{1}(t_{n}),\qquad U_{M}^{n}=A_{2}(t_{n}),\quad 1\leq n \leq N. \end{cases} $$

Consider the perturbed system of (3.1) with perturbation in the initial values

3.2 $$ \textstyle\begin{cases} \wedge _{1} \hat{U}_{j}^{n}+i\mu \wedge \hat{U}_{j}^{n}+\lambda \kappa \wedge \hat{U}_{j}^{n} \\ \quad =i\mu \wedge \hat{U}_{j}^{n-1}-i\mu \sum_{k=0}^{n-2}R_{n,k}^{ \gamma }(\wedge \hat{U}_{j}^{k+1}-\wedge \hat{U}_{j}^{k})+\wedge f_{j}^{n},\quad 1\leq j\leq M-1, \\ \hat{U}_{j}^{0}=\hat{A}_{0}(x_{j}), \quad 0\leq j\leq M, \\ \hat{U}_{0}^{n}=A_{1}(t_{n}), \qquad \hat{U}_{M}^{n}=A_{2}(t_{n}),\quad 1\leq n\leq N. \end{cases} $$

Let $U_{j}^{n}$ be the solution of (3.1) and let $\hat{U}_{j}^{n}$ be the solution of the perturbed system (3.2). Let $\rho _{j}^{n}={U}_{j}^{n}-\hat{U}_{j}^{n}$, $0\leq j\leq M$, $0 \leq n\leq N$. It follows from (3.1) and (3.2) that

3.3 $$ \textstyle\begin{cases} \wedge _{1} \rho _{j}^{n}+i\mu \wedge \rho _{j}^{n}+\lambda \kappa \wedge \rho _{j}^{n} \\ \quad =i\mu \wedge \rho _{j}^{n-1}-i\mu \sum_{k=0}^{n-2}R_{n,k}^{ \gamma }(\wedge \rho _{j}^{k+1}-\wedge \rho _{j}^{k}),\quad 1\leq j \leq M-1, \\ \rho _{j}^{0}= A_{0}(x_{j})-\hat{A}_{0}(x_{j}),\quad 0\leq j\leq M, \\ \rho _{0}^{n}=0, \qquad \rho _{M}^{n}=0,\quad 1\leq n\leq N. \end{cases} $$

Denote $\rho ^{n}=[\rho _{0}^{n},\rho _{1}^{n},\ldots ,\rho _{M}^{n}]$, $0 \leq n\leq N$. Since $\rho _{0}^{n}=\rho _{M}^{n}=0$, we define the *L*2 norm of $\rho ^{n}$ by

3.4 $$ \bigl\Vert \rho ^{n} \bigr\Vert _{2} = \Biggl(\sum _{j=1}^{M-1}h \bigl\vert \rho _{j}^{n} \bigr\vert ^{2} \Biggr)^{\frac{1}{2}}. $$

To apply the Fourier method, we expand $\rho ^{n}$ to a piecewise constant function $\rho ^{n}(x)$, where

3.5 $$ \rho ^{n}(x)= \textstyle\begin{cases} \displaystyle \rho _{j}^{n}, & \displaystyle x\in \bigg(x_{j}-\frac{h}{2}, x_{j}+ \frac{h}{2} \bigg], 1\leq j\leq M-1, \\ \displaystyle 0, & \displaystyle x\in \bigg[0,\frac{h}{2} \bigg] \cup \bigg(L- \frac{h}{2}, L \bigg]. \end{cases} $$

Then $\rho ^{n}(x)$ can be expanded as a Fourier series,

3.6 $$ \rho ^{n}(x)=\sum_{m=-\infty }^{\infty }d_{n}(m)e^{i2\pi mx/L},\quad 0\leq n\leq N, $$

where

3.7 $$ d_{n}(m)=\frac{1}{L} \int _{0}^{L} \rho ^{n}(x)e^{-i2\pi mx/L} \,dx. $$

To carry out the Fourier stability analysis, it is sufficient to consider an individual harmonic of the form [6]

$$ \rho _{j}^{n}(m)=d_{n}(m)e^{i2\pi m jh/L}. $$

### Lemma 3.1

*Suppose the solution of* (3.3) *has the following form*:

3.8 $$ \rho _{j}^{n}(m)=d_{n}(m)e^{i\theta jh}, $$

*where* $\theta =2\pi m/L$ *and* *m* *is the wave number*. *Then the following inequality holds*:

3.9 $$ \bigl\vert d_{n}(m) \bigr\vert \leq \bigl\vert d_{0}(m) \bigr\vert , \quad 1\leq n\leq N. $$

### Proof

For notational simplicity, let $d_{n}\equiv d_{n}(m)$ for a fixed wave number *m*. We shall use mathematical induction to complete the proof. First, we consider $n=1$. Upon substituting (3.8) into (3.3), we obtain

3.10 $$ \wedge _{1} d_{1}e^{i\theta jh}+i\mu \wedge d_{1}e^{i\theta jh}+ \lambda \kappa \wedge d_{1}e^{i\theta jh}=i \mu \wedge d_{0}e^{i \theta jh}. $$

After a series of computations, (3.10) leads to

3.11 $$ d_{1}=\Omega d_{0}, $$

where

3.12 $$\begin{aligned}& \Omega =\frac{i\eta _{j} }{\phi _{j}+i\eta _{j} }, \end{aligned}$$

3.13 $$\begin{aligned}& \eta _{j}= \textstyle\begin{cases} 2b_{0}\mu \cos (2\theta h)+2b_{1}\mu \cos (\theta h)+b_{2}\mu , & j=1, M-1, \\ 2p\mu \cos (2\theta h)+2q\mu \cos (\theta h)+s\mu , & 2\leq j\leq M-2, \end{cases}\displaystyle \end{aligned}$$

and

3.14 $$ \phi _{j}= \textstyle\begin{cases} \displaystyle \biggl[ \frac{2a_{0}}{h^{2}}+2b_{0}\lambda \kappa \biggr]\cos (2\theta h)+ \biggl[\frac{2a_{1}}{h^{2}}+2b_{1}\lambda \kappa \biggr]\cos (\theta h) \\ \displaystyle \quad {}+\frac{a_{2}}{h^{2}}+b_{2}\lambda \kappa , & j=1, M-1, \\ \displaystyle \biggl[ \frac{2\alpha }{h^{2}}+2p\lambda \kappa \biggr] \cos (2\theta h)+ \biggl[\frac{4(\beta -\alpha )}{h^{2}}+2q\lambda \kappa \biggr]\cos (\theta h) \\ \displaystyle \quad {}+\frac{2\alpha -4\beta }{h^{2}}+s \lambda \kappa , & 2\leq j\leq M-2. \end{cases} $$

It is obvious that $|\Omega |\leq 1$, therefore from (3.11) we have

3.15 $$ \vert d_{1} \vert \leq \vert d_{0} \vert . $$

Next, assume that $|d_{\ell }|\leq |d_{0}|$, $1\leq \ell \leq n-1$. We shall show that $|d_{n}| \leq |d_{0}|$. For any $n\geq 2$, we substitute (3.8) into (3.3) to get

3.16 $$ \begin{aligned} &\wedge _{1} d_{n}e^{i\theta jh}+i\mu \wedge d_{n}e^{i\theta jh}+ \lambda \kappa \wedge d_{n}e^{i\theta jh} \\ &\quad =i \mu \wedge d_{n-1}e^{i \theta jh}-i\mu \sum _{k=0}^{n-2}R_{n,k}^{\gamma } \bigl(\wedge d_{k+1}e^{i \theta jh}-\wedge d_{k}e^{i\theta jh} \bigr). \end{aligned} $$

After a series of computations, (3.16) yields

$$ d_{n}=\Omega d_{n-1}- \Omega \sum _{k=0}^{n-2} R_{n,k}^{\gamma }(d_{k+1}-d_{k}), $$

or equivalently

3.17 $$ d_{n}=\Omega \Biggl[ \bigl(1-R_{n,n-2}^{\gamma } \bigr)d_{n-1}+\sum_{k=1}^{n-2} \bigl(R_{n,k}^{\gamma }-R_{n,k-1}^{\gamma } \bigr)d_{k}+R_{n,0}^{ \gamma }d_{0} \Biggr]. $$

Using Lemma 2.3, $|d_{\ell }|\leq |d_{0}|$ for $1\leq \ell \leq n-1$, and $|\Omega |\leq 1$, it follows from (3.17) that

3.18 $$ \begin{aligned} \vert d_{n} \vert &= \vert \Omega \vert \Biggl\vert \bigl(1-R_{n,n-2}^{\gamma } \bigr)d_{n-1}+\sum_{k=1}^{n-2} \bigl(R_{n,k}^{\gamma }-R_{n,k-1}^{ \gamma } \bigr)d_{k}+R_{n,0}^{\gamma }d_{0} \Biggr\vert \\ &\leq \vert \Omega \vert \Biggl[ \bigl\vert \bigl(1-R_{n,n-2}^{ \gamma } \bigr) \bigr\vert \vert d_{n-1} \vert +\sum _{k=1}^{n-2} \bigl\vert \bigl(R_{n,k}^{ \gamma }-R_{n,k-1}^{\gamma } \bigr) \bigr\vert \vert d_{k} \vert + \bigl\vert R_{n,0}^{ \gamma } \bigr\vert \vert d_{0} \vert \Biggr] \\ &\leq \vert \Omega \vert \Biggl[ \bigl(1-R_{n,n-2}^{\gamma } \bigr) \vert d_{0} \vert +\sum_{k=1}^{n-2} \bigl(R_{n,k}^{\gamma }-R_{n,k-1}^{ \gamma } \bigr) \vert d_{0} \vert +R_{n,0}^{\gamma } \vert d_{0} \vert \Biggr] \\ &= \vert \Omega \vert \vert d_{0} \vert \Biggl[ \bigl(1-R_{n,n-2}^{\gamma } \bigr)+\sum _{k=1}^{n-2} \bigl(R_{n,k}^{\gamma }-R_{n,k-1}^{\gamma } \bigr)+R_{n,0}^{\gamma } \Biggr] \\ &= \vert \Omega \vert \vert d_{0} \vert \leq \vert d_{0} \vert . \end{aligned} $$

Hence, we have completed the proof of (3.9). □

### Theorem 3.1

(Stability)

*The numerical scheme* (2.25)*–*(2.27) *or equivalently* (3.1) *is unconditionally stable with respect to the initial data*.

### Proof

From the definition of the *L*2 norm (3.4) and (3.5), we find

3.19 $$ \begin{aligned} \bigl\Vert \rho ^{n} \bigr\Vert _{2} &= \Biggl( \sum_{j=1}^{M-1} h \bigl\vert \rho _{j}^{n} \bigr\vert ^{2} \Biggr)^{\frac{1}{2}} \\ &= \Biggl[ \int _{0}^{h/2} \bigl\vert \rho ^{n}(x) \bigr\vert ^{2}\,dx+ \sum _{j=1}^{M-1} \int _{x_{j}-h/2}^{x_{j}+h/2} \bigl\vert \rho ^{n}(x) \bigr\vert ^{2}\,dx+ \int _{L-h/2}^{L} \bigl\vert \rho ^{n}(x) \bigr\vert ^{2}\,dx \Biggr]^{\frac{1}{2}} \\ &= \biggl[ \int _{0}^{L} \bigl\vert \rho ^{n}(x) \bigr\vert ^{2} \,dx \biggr]^{ \frac{1}{2}}. \end{aligned} $$

Noting the Parseval equality [8, 10]

3.20 $$ \int _{0}^{L} \bigl\vert \rho ^{n}(x) \bigr\vert ^{2} \,dx=\sum _{m=-\infty }^{ \infty } \bigl\vert d_{n}(m) \bigr\vert ^{2}, $$

it follows from (3.19) that

3.21 $$ \bigl\Vert \rho ^{n} \bigr\Vert _{2}^{2}= \sum_{m=-\infty }^{\infty } \bigl\vert d_{n}(m) \bigr\vert ^{2}. $$

Applying Lemma 3.1 in (3.21), we obtain

3.22 $$ \bigl\Vert \rho ^{n} \bigr\Vert _{2}^{2}= \sum_{m=-\infty }^{\infty } \bigl\vert d_{n}(m) \bigr\vert ^{2} \leq \sum _{m=-\infty }^{\infty } \bigl\vert d_{0}(m) \bigr\vert ^{2}= \bigl\Vert \rho ^{0} \bigr\Vert _{2}^{2},\quad 1\leq n\leq N, $$

which shows that the numerical scheme (3.1) is robust to perturbation of initial data. □

## Solvability of the numerical scheme

In this section, we shall investigate the solvability of the numerical scheme (2.25)–(2.27) or equivalently (3.1). It is clear that the corresponding homogeneous system of (3.1) is

4.1 $$ \textstyle\begin{cases} \wedge _{1} U_{j}^{n}+i\mu \wedge U_{j}^{n}+\lambda \kappa \wedge U_{j}^{n} \\ \quad =i \mu \wedge U_{j}^{n-1}-i\mu \sum_{k=0}^{n-2}R_{n,k}^{\gamma }(\wedge U_{j}^{k+1}- \wedge U_{j}^{k}), \quad 1\leq j\leq M-1, \\ U_{j}^{0}=0, \quad 0\leq j\leq M, \\ U_{0}^{n}=0,\qquad U_{M}^{n}=0,\quad 1\leq n\leq N. \end{cases} $$

Similar to the proof of Theorem 3.1, we can verify that the solution of (4.1) satisfies

$$ \bigl\Vert U^{n} \bigr\Vert _{2}\leq \bigl\Vert U^{0} \bigr\Vert _{2},\quad 1\leq n\leq N. $$

Since $U^{0}=0$, it follows that $U^{n}=0$ for $1\leq n\leq N$. Hence, (4.1) has only the trivial solution and we obtain the following theorem.

### Theorem 4.1

(Solvability)

*The numerical scheme* (2.25)*–*(2.27) *or equivalently* (3.1) *is uniquely solvable*.

## Convergence of the numerical scheme

In this section, we shall establish the convergence of the numerical scheme (2.25)–(2.27) or equivalently (3.1). Recall that $u(x,t)$ is the exact solution of (1.1) and $U_{j}^{n}$ is the numerical approximation of $u_{j}^{n}$ obtained from (3.1). Let the error $E_{j}^{n}$ at $(x_{j},t_{n})$ be $E_{j}^{n}=u_{j}^{n}-U_{j}^{n}$, $0\leq j\leq M$, $0\leq n\leq N$.

Clearly, from (3.1) we obtain the error equation

5.1 $$ \begin{aligned} &\wedge _{1} E_{j}^{n}+i \mu \wedge E_{j}^{n}+\lambda \kappa \wedge E_{j}^{n}=i \mu \wedge E_{j}^{n-1}-i \mu \sum_{k=0}^{n-2}R_{n,k}^{\gamma } \bigl(\wedge E_{j}^{k+1}- \wedge E_{j}^{k} \bigr)+T_{j}^{n}, \\ &\quad 1\leq j\leq M-1, 1\leq n\leq N, \end{aligned} $$

where $T_{j}^{n}$ is the local truncation error,

$$ E_{j}^{n}=0, \quad 0\leq j\leq M \quad \mbox{and}\quad E_{0}^{n}=E_{M}^{n}=0, \quad 1\leq n\leq N. $$

Denote $E^{n}=[E_{0}^{n},E_{1}^{n},\ldots ,E_{M}^{n}]$, $0\leq n\leq N$. Since $E_{0}^{n}=E_{M}^{n}=0$, we define the *L*2 norm of $E^{n}$ by

$$ \bigl\Vert E^{n} \bigr\Vert _{2} = \Biggl(\sum _{j=1}^{M-1}h \bigl\vert E_{j}^{n} \bigr\vert ^{2} \Biggr)^{ \frac{1}{2}}. $$

Likewise, denote $T^{n}=[T_{1}^{n},T_{2}^{n},\ldots ,T_{M-1}^{n}]$, $1\leq n\leq N$ and the *L*2 norm of $T^{n}$ is given by

$$ \bigl\Vert T^{n} \bigr\Vert _{2} = \Biggl(\sum _{j=1}^{M-1}h \bigl\vert T_{j}^{n} \bigr\vert ^{2} \Biggr)^{ \frac{1}{2}}. $$

In view of (2.16), (2.17) and (2.22), we see that $T_{j}^{n}=O(h^{6}+\tau ^{2-\gamma })$ and there is a constant $C_{1}$ such that

$$ \bigl\vert T_{j}^{n} \bigr\vert \leq C_{1} \bigl(h^{6}+\tau ^{2-\gamma } \bigr),\quad 1\leq j \leq M-1, 1\leq n\leq N. $$

It follows that

5.2 $$ \bigl\Vert T^{n} \bigr\Vert _{2}= \Biggl(\sum _{j=1}^{M-1}h \bigl\vert T_{j}^{n} \bigr\vert ^{2} \Biggr)^{ \frac{1}{2}}\leq C_{1}\sqrt{L} \bigl(h^{6}+ \tau ^{2-\gamma } \bigr). $$

Next, similar to (3.5)–(3.7), we define the piecewise constant functions $E^{n}(x)$ and $T^{n}(x)$ by

5.3 $$ E^{n}(x)= \textstyle\begin{cases} E_{j}^{n}, & \displaystyle x\in \bigg(x_{j}-\frac{h}{2}, x_{j}+ \frac{h}{2} \bigg], 1\leq j\leq M-1, \\ 0, & \displaystyle x\in \biggl[0,\frac{h}{2} \biggr] \cup \bigg(L- \frac{h}{2}, L \bigg], \end{cases} $$

and

5.4 $$ T^{n}(x)= \textstyle\begin{cases} T_{j}^{n}, & \displaystyle x\in \bigg(x_{j}-\frac{h}{2}, x_{j}+ \frac{h}{2} \bigg], 1\leq j\leq M-1, \\ 0, & \displaystyle x\in \biggl[0,\frac{h}{2} \biggr] \cup \bigg(L- \frac{h}{2}, L \bigg]. \end{cases} $$

Then $E^{n}(x)$ and $T^{n}(x)$ have the Fourier series expansions

5.5 $$ E^{n}(x)=\sum_{m=-\infty }^{\infty } \epsilon _{n}(m)e^{i2\pi mx/L} ,\qquad T^{n}(x)=\sum _{m=-\infty }^{\infty }\delta _{n}(m)e^{i2\pi mx/L} , $$

where

5.6 $$ \epsilon _{n}(m)=\frac{1}{L} \int _{0}^{L} E^{n}(x)e^{-i2\pi mx/L} \,dx, \qquad \delta _{n}(m)=\frac{1}{L} \int _{0}^{L} T^{n}(x)e^{-i2\pi mx/L} \,dx. $$

Similar to (3.19)–(3.21), by applying the Parseval identities we find

5.7 $$ \bigl\Vert E^{n} \bigr\Vert _{2}^{2}= \sum_{m=-\infty }^{\infty } \bigl\vert \epsilon _{n}(m) \bigr\vert ^{2},\qquad \bigl\Vert T^{n} \bigr\Vert _{2}^{2}=\sum _{m=-\infty }^{\infty } \bigl\vert \delta _{n}(m) \bigr\vert ^{2}. $$

Further, noting (5.2), we see that $\sum_{m=-\infty }^{\infty }|\delta _{n}(m)|^{2}$ converges and there is a positive constant $C_{2}\geq 1$ [7] such that

5.8 $$ \bigl\vert \delta _{n}(m) \bigr\vert \leq C_{2} \bigl\vert \delta _{1}(m) \bigr\vert ,\quad 1\leq n\leq N. $$

As in Sect. 3, we shall consider individual harmonics $E_{j}^{n}(m)$ and $T_{j}^{n}(m)$ of the forms

5.9 $$ E_{j}^{n}(m)=\epsilon _{n}(m)e^{i\theta jh},\qquad T_{j}^{n}(m)= \delta _{n}(m)e^{i\theta jh}, $$

where $\theta =2\pi m/L$. Denote $\epsilon _{n}\equiv \epsilon _{n}(m)$ and $\delta _{n}\equiv \delta _{n}(m)$ for a fixed wave number *m*. Upon substituting (5.9) into (5.1), we get

5.10 $$ \begin{aligned} &{}\wedge _{1} \epsilon _{n}e^{i\theta jh}+i\mu \wedge \epsilon _{n}e^{i \theta jh}+ \lambda \kappa \wedge \epsilon _{n}e^{i\theta jh} \\ &\quad =i\mu \wedge \epsilon _{n-1}e^{i\theta jh}-i\mu \sum _{k=0}^{n-2}R_{n,k}^{ \gamma } \bigl(\wedge \epsilon _{k+1}e^{i\theta jh}-\wedge \epsilon _{k}e^{i \theta jh} \bigr)+\delta _{n}e^{i\theta jh}, \\ &\qquad 1\leq j\leq M-1, 1 \leq n\leq N. \end{aligned} $$

Now, we shall present two lemmas which are essential in the proof of the convergence result.

### Lemma 5.1

*Let* $\alpha \in (0,\frac{1}{10} )\cup (\frac{1}{10}, \frac{37}{180} )$. *Then we have*

5.11 $$ \vert s \vert -2 \vert p \vert -2 \vert q \vert >0, $$

*where* *p*, *q* *and* *s* *are defined in* (2.10).

### Proof

Recall from Remark 2.2 that $\alpha \neq \frac{1}{10}$. For $\alpha \in (0,\frac{1}{20} )$, we have $p<0$, $q>0$ and $s>0$, therefore

$$ \vert s \vert -2 \vert p \vert -2 \vert q \vert = - \frac{3}{2}\alpha +\frac{97}{120}-2 \biggl( \frac{1}{240}- \frac{1}{12}\alpha \biggr)-2 \biggl( \frac{2}{3} \alpha + \frac{1}{10} \biggr) = - \frac{8}{3}\alpha + \frac{3}{5}>0. $$

For $\alpha \in [\frac{1}{20},\frac{1}{10} )\cup ( \frac{1}{10},\frac{37}{180} )$, we have $p\geq 0$, $q>0$ and $s>0$, therefore

$$ \vert s \vert -2 \vert p \vert -2 \vert q \vert = - \frac{3}{2}\alpha +\frac{97}{120}-2 \biggl( \frac{1}{12} \alpha -\frac{1}{240} \biggr)-2 \biggl( \frac{2}{3} \alpha + \frac{1}{10} \biggr) = -{3}\alpha + \frac{37}{60}>0. $$

The proof is completed. □

### Lemma 5.2

*Let* $\alpha \in (0,\frac{1}{10} )\cup (\frac{1}{10}, \frac{37}{180} )$. *Let* $\epsilon _{n}$, $1\leq n\leq N$ *be the solution of* (5.10). *Then we have*

5.12 $$ \vert \epsilon _{n} \vert \leq C_{2}C_{3} \vert \delta _{1} \vert ,\quad 1\leq n\leq N, $$

*where* $C_{3}=T^{\gamma }\Gamma (1-\gamma )/\overline{C}$ *and* $\overline{C}=\min \{ \frac{19}{60}, |s|-2|p|-2|q| \} $.

### Proof

Since $E^{0}=0$, it is clear from (5.7) that

5.13 $$ \epsilon _{0}(m)=\epsilon _{0}=0. $$

We shall first show that (5.12) is true for $n=1$. Indeed, when $n=1$, from (5.10) we get

5.14 $$ \epsilon _{1}=\frac{1}{\phi _{j}+i\eta _{j}}\delta _{1}, $$

where $\eta _{j}$ and $\phi _{j}$ are defined in (3.13) and (3.14), respectively.

Next, it is clear from Lemma 5.1 that $\overline{C}>0$. Moreover, we note that $|b_{2}|-2|b_{0}|-2|b_{1}|=\frac{19}{60}$, where $b_{0}$, $b_{1}$ and $b_{2}$ are given in (2.15). Together with (3.13), we obtain

5.15 $$ \begin{aligned} \overline{C}&= \min \biggl\{ \frac{19}{60}, \vert s \vert -2 \vert p \vert -2 \vert q \vert \biggr\} \\ &= \min \bigl\{ \vert b_{2} \vert -2 \vert b_{0} \vert -2 \vert b_{1} \vert , \vert s \vert -2 \vert p \vert -2 \vert q \vert \bigr\} \\ &\leq \min \bigl\{ \bigl\vert b_{2}+2b_{0}\cos {(2 \theta h)}+2b_{1} \cos {(\theta h)} \bigr\vert , \bigl\vert s+2p \cos {(2\theta h)}+2q\cos {( \theta h)} \bigr\vert \bigr\} \\ &\leq \frac{1}{\mu } \vert \eta _{j} \vert ,\quad 1\leq j\leq M-1. \end{aligned} $$

Using (5.15), the fact $|\Omega |= \vert \frac{i\eta _{j}}{\phi _{j}+i\eta _{j}} \vert \leq 1$, the definition of *μ* (Lemma 2.2), Lemma 2.3 and $C_{2}\geq 1$ (refer to (5.8)), we further find from (5.14)

5.16 $$\begin{aligned} \vert \epsilon _{1} \vert &= \frac{1}{ \vert \phi _{j}+i\eta _{j} \vert } \vert \delta _{1} \vert \\ &= \frac{C_{3}\overline{C}}{T^{\gamma }\Gamma (1-\gamma )} \frac{1}{ \vert \phi _{j}+i\eta _{j} \vert } \vert \delta _{1} \vert \\ &\leq \frac{C_{3}}{T^{\gamma }\Gamma (1-\gamma )} \frac{ \vert i\eta _{j} \vert }{\mu } \frac{1}{ \vert \phi _{j}+i\eta _{j} \vert } \vert \delta _{1} \vert \\ &= \frac{\tau ^{\gamma }\Gamma (2-\gamma )C_{3}}{T^{\gamma }\Gamma (1-\gamma )} \vert \Omega \vert \vert \delta _{1} \vert \\ &= \frac{1-\gamma }{N^{\gamma }}C_{3} \vert \Omega \vert \vert \delta _{1} \vert < R_{N,0}^{\gamma }C_{3} \vert \Omega \vert \vert \delta _{1} \vert \leq C_{2}C_{3} \vert \delta _{1} \vert . \end{aligned}$$

Hence, we have proved (5.12) for $n=1$.

Now, assume that, for $n\geq 2$,

5.17 $$ \vert \epsilon _{\ell } \vert \leq C_{2}C_{3} \vert \delta _{1} \vert ,\quad 1\leq \ell \leq n-1. $$

From (5.10), after a series of computations we get

$$ \epsilon _{n}=\Omega \epsilon _{n-1}- \Omega \sum _{k=0}^{n-2} R_{n,k}^{ \gamma }( \epsilon _{k+1}-\epsilon _{k})+\frac{1}{\phi _{j}+i\eta _{j}} \delta _{n}, $$

or equivalently

5.18 $$ \epsilon _{n}=\Omega \Biggl[ \bigl(1-R_{n,n-2}^{\gamma } \bigr) \epsilon _{n-1}+\sum_{k=1}^{n-2} \bigl(R_{n,k}^{\gamma }-R_{n,k-1}^{ \gamma } \bigr)\epsilon _{k}+R_{n,0}^{\gamma }\epsilon _{0} \Biggr] + \frac{1}{\phi _{j}+i\eta _{j}}\delta _{n}. $$

Using (5.13), by a similar argument to (5.16), (5.8), Lemma 2.3, (5.17) and $|\Omega |\leq 1$, it follows from (5.18) that

$$ \begin{aligned} \vert \epsilon _{n} \vert &\leq \vert \Omega \vert \Biggl\vert \bigl(1-R_{n,n-2}^{ \gamma } \bigr)\epsilon _{n-1}+\sum_{k=1}^{n-2} \bigl(R_{n,k}^{ \gamma }-R_{n,k-1}^{\gamma } \bigr)\epsilon _{k}+R_{n,0}^{\gamma } \epsilon _{0} \Biggr\vert +\frac{1}{ \vert \phi _{j}+i\eta _{j} \vert } \vert \delta _{n} \vert \\ &\leq \vert \Omega \vert \Biggl[ \bigl\vert \bigl(1-R_{n,n-2}^{ \gamma } \bigr)\epsilon _{n-1} \bigr\vert + \Biggl\vert \sum _{k=1}^{n-2} \bigl(R_{n,k}^{ \gamma }-R_{n,k-1}^{\gamma } \bigr)\epsilon _{k} \Biggr\vert \Biggr]+R^{\gamma }_{n,0}C_{3} \vert \Omega \vert \vert \delta _{n} \vert \\ &\leq \vert \Omega \vert \Biggl[ \bigl\vert \bigl(1-R_{n,n-2}^{ \gamma } \bigr) \bigr\vert \vert \epsilon _{n-1} \vert +\sum _{k=1}^{n-2} \bigl\vert \bigl(R_{n,k}^{\gamma }-R_{n,k-1}^{\gamma } \bigr) \bigr\vert \vert \epsilon _{k} \vert +R^{\gamma }_{n,0}C_{2}C_{3} \vert \delta _{1} \vert \Biggr] \\ &\leq C_{2}C_{3} \vert \Omega \vert \vert \delta _{1} \vert \Biggl[ \bigl(1-R_{n,n-2}^{ \gamma } \bigr)+\sum_{k=1}^{n-2} \bigl(R_{n,k}^{\gamma }-R_{n,k-1}^{ \gamma } \bigr)+R^{\gamma }_{n,0} \Biggr] \\ &\leq C_{2}C_{3} \vert \delta _{1} \vert . \end{aligned} $$

Hence, we have proved (5.12). □

### Theorem 5.1

(Convergence)

*Let* $\alpha \in (0,\frac{1}{10} )\cup (\frac{1}{10}, \frac{37}{180} )$. *Suppose* $u(x,t)$ *is the exact solution of* (1.1) *and* $u(x,t)\in C^{(8,2)}([0,L]\times [0,T])$. *Then we have*, *for* $1\leq n\leq N$,

5.19 $$ \bigl\Vert E^{n} \bigr\Vert _{2}=O \bigl(h^{6}+\tau ^{2-\gamma } \bigr). $$

*Hence*, *the numerical scheme* (2.25)*–*(2.27) *or equivalently* (3.1) *is convergent with order* $O(h^{6}+\tau ^{2-\gamma })$.

### Proof

Using (5.7), Lemma 5.2 and (5.2), we find, for $1\leq n\leq N$,

$$ \begin{aligned} \bigl\Vert E^{n} \bigr\Vert _{2}&= \Biggl(\sum_{m=-\infty }^{\infty } \bigl\vert \epsilon _{n}(m) \bigr\vert ^{2} \Biggr)^{\frac{1}{2}} \\ &\leq C_{2}C_{3} \Biggl(\sum _{m=-\infty }^{\infty } \bigl\vert \delta _{1}(m) \bigr\vert ^{2} \Biggr)^{\frac{1}{2}} \\ &= C_{2}C_{3} \bigl\Vert T^{1} \bigr\Vert _{2} \\ &\leq C_{1}C_{2}C_{3}\sqrt{L} \bigl(h^{6}+\tau ^{2-\gamma } \bigr). \end{aligned} $$

The proof is completed. □

### Remark 5.1

As shown in Theorem 5.1, the numerical scheme (2.25)–(2.27) achieves sixth order convergence in the spatial dimension. This *improves* the work of [36] where the spatial convergence order is observed to be four through numerical experiment. Furthermore, in [36] the authors have only proved the stability of their scheme, while we have proven the stability, unique solvability and convergence of our method by the Fourier method. We remark that the Fourier method is *rarely* used in the analysis of numerical methods of time/space-fractional Schrödinger equation, especially in establishing the convergence order, so we have successfully illustrated the analytical technique of the Fourier method in this work. It is noted that the energy method has been commonly used to show the convergence of numerical methods for the space-fractional Schrödinger equation [31, 62–65].

## Numerical examples

In this section, we shall present three numerical examples to verify the efficiency of the scheme (2.25)–(2.27) and to compare with other methods in the literature.

Note that our theoretical convergence result is in *L*2 norm, nonetheless it would also be interesting to see the convergence in maximum norm. In fact, for a fixed pair of $(h,\tau )$, we shall compute the following accuracy indicators: maximum *L*2 norm error $E_{2}(h,\tau )$, maximum modulus error $E_{\infty }(h,\tau )$, maximum real part absolute error $E_{\infty }^{\mathrm{Re}}(h,\tau )$ and maximum imaginary part absolute error $E_{\infty }^{\mathrm{Im}}(h,\tau )$, defined by

6.1 $$\begin{aligned}& E_{2}(h,\tau )= \max_{0\leq n\leq N} \bigl\Vert E^{n} \bigr\Vert _{2} = \max_{0\leq n \leq N} \Biggl(\sum_{j=1}^{M-1}h \bigl\vert u_{j}^{n}-U_{j}^{n} \bigr\vert ^{2} \Biggr)^{ \frac{1}{2}}, \end{aligned}$$

6.2 $$\begin{aligned}& E_{\infty }(h,\tau )= \max_{0\leq n\leq N}\max _{0\leq j \leq M} \bigl\vert u_{j}^{n}-U_{j}^{n} \bigr\vert , \end{aligned}$$

6.3 $$\begin{aligned}& \begin{aligned} & E_{\infty }^{\mathrm{Re}}(h,\tau )= \max_{0\leq n\leq N}\max _{0 \leq j \leq M} \bigl\vert \mathrm{Re} \bigl(u_{j}^{n}-U_{j}^{n} \bigr) \bigr\vert , \\ & E_{\infty }^{\mathrm{Im}}(h, \tau )= \max _{0\leq n\leq N} \max_{0\leq j \leq M} \bigl\vert \mathrm{Im} \bigl(u_{j}^{n}-U_{j}^{n} \bigr) \bigr\vert . \end{aligned} \end{aligned}$$

The convergence orders in temporal and spatial dimensions can be computed by

6.4 $$\begin{aligned}& \mbox{Temporal}_{2}=\log _{2} \biggl[ \frac{E_{2}(h,2\tau )}{E_{2}(h,\tau )} \biggr],\qquad \mbox{Temporal}_{ \infty }=\log _{2} \biggl[ \frac{E_{\infty }(h,2\tau )}{E_{\infty }(h,\tau )} \biggr], \end{aligned}$$

6.5 $$\begin{aligned}& \mbox{Space}_{2}=\log _{2} \biggl[ \frac{E_{2}(2h,\tau )}{E_{2}(h,\tau )} \biggr],\qquad \mbox{Space}_{ \infty }=\log _{2} \biggl[ \frac{E_{\infty }(2h,\tau )}{E_{\infty }(h,\tau )} \biggr]. \end{aligned}$$

### Example 6.1

([16, 36, 47])

Consider the time-fractional Schrödinger equation

6.6 $$ \textstyle\begin{cases}\displaystyle i\,{}^{C}_{0}{D}^{\gamma }_{t} u(x,t)+ \frac{\partial ^{2} u(x,t)}{\partial x^{2}}+ \big\vert u(x,t) \big\vert ^{2}u(x,t)=f(x,t), \quad (x,t)\in [0,1]\times [0,1], \\ u(x,0)=0, \quad x\in [0,1], \\ u(0,t)=it^{2}, \qquad u(1,t)=it^{2}, \quad t\in [0,1], \end{cases} $$

where $0<\gamma <1$ and

6.7 $$ \begin{aligned} f(x,t)&= -\frac{2t^{2-\gamma }}{\Gamma (3-\gamma )}\cos (2 \pi x)+ \bigl(-4\pi ^{2} t^{2}+t^{6} \bigr)\sin (2\pi x) \\ &\quad {} + i \biggl[\frac{2t^{2-\gamma }}{\Gamma (3-\gamma )} \sin (2\pi x)+ \bigl(-4\pi ^{2}t^{2}+t^{6} \bigr)\cos (2\pi x) \biggr]. \end{aligned} $$

The exact solution is $u(x,t)=t^{2}[\sin (2\pi x)+i\cos (2\pi x)]$.

**Figure 1:**
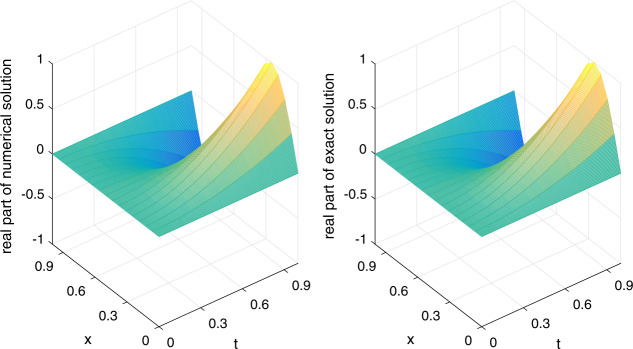
(Example 6.1) Real part of numerical solution and exact solution

**Figure 2:**
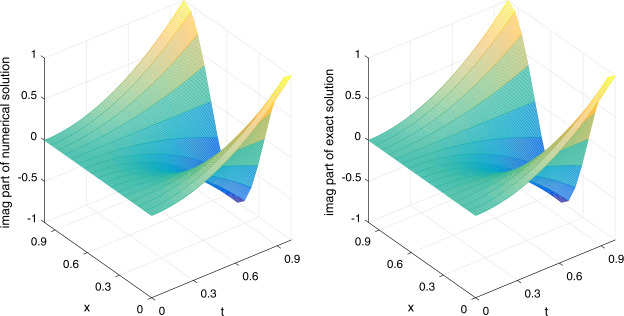
(Example 6.1) Imaginary part of numerical solution and exact solution
(vi) In Fig. 3, we plot the absolute modulus error $|u_{j}^{n}-U_{j}^{n}|$ obtained from the scheme (2.25)–(2.27) for $\gamma =0.1$ and $(h,\tau )=(1/16,1/200)$. We observe from Fig. 3 that the error is very small.

**Figure 3:**
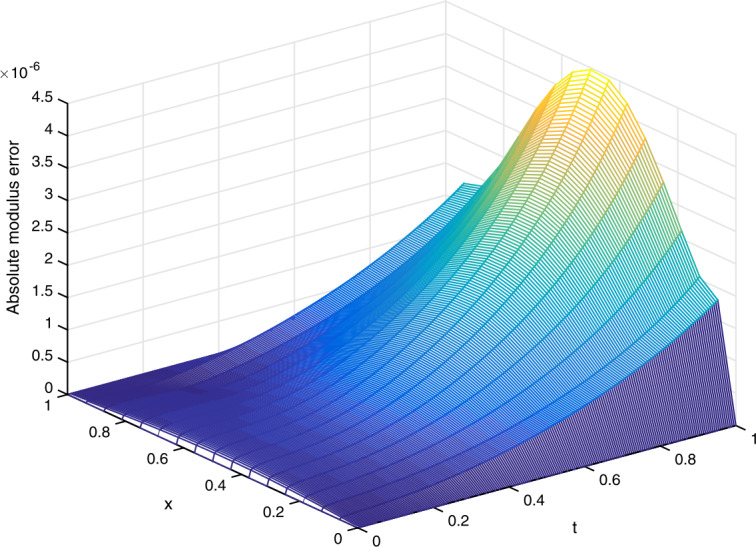
(Example 6.1) Absolute modulus error

Let $\alpha =1/20$ be fixed. We shall apply the scheme (2.25)–(2.27) to compute the errors and convergence orders, and comparisons are made with other methods in the literature. There follows a brief description of the numerical simulation in Tables 1–4 and Figs. 1–3:

(i) In Table 1, we fix $h=1/1000$ and let *τ* vary. Applying (2.25)–(2.27), we compute $E_{2}(h,\tau )$, $E_{\infty }(h,\tau )$ and the respective temporal convergence orders. The numerical results indicate that our method is of order $(2-\gamma )$ in the temporal dimension, thus verifying the theoretical temporal convergence order in Theorem 5.1.

**Table 1:** (Example 6.1) $E_{2}(h,\tau )$, $E_{\infty }(h,\tau )$ and temporal convergence orders

*γ*	*τ*	$E_{2}(h,\tau )$	Temporal_2_	$E_{\infty }(h,\tau )$	$\text{Temporal}_{\infty }$
0.2	1/20	3.4967e−05		5.0107e−05	
	1/40	1.0627e−05	1.7183	1.5231e−05	1.7180
	1/80	3.2052e−06	1.7292	4.5947e−06	1.7290
0.4	1/20	1.2356e−04		1.7701e−04	
	1/40	4.1683e−05	1.5677	5.9711e−05	1.5678
	1/80	1.3977e−05	1.5764	2.0021e−05	1.5765
0.6	1/20	3.4186e−04		4.8943e−04	
	1/40	1.3047e−04	1.3897	1.8678e−04	1.3898
	1/80	4.9681e−05	1.3930	7.1121e−05	1.3930
(ii) In Table 2, we fix $\tau =1/5000$ and let *h* vary. We present $E_{2}(h,\tau )$, $E_{\infty }(h,\tau )$ and the spatial convergence orders of our scheme (2.25)–(2.27) and those of the cubic non-polynomial spline (CNS) method [36]. From Table 2, we see that our method can achieve at least $O(h^{6})$, while the CNS method is $O(h^{4})$. Moreover, our method obtains smaller errors in all the cases. The observation also confirms the theoretical spatial convergence order in Theorem 5.1.

**Table 2:** (Example 6.1) $E_{2}(h,\tau )$, $E_{\infty }(h,\tau )$ and spatial convergence orders

*γ*	*h*	$E_{2}(h,\tau )$	Space_2_	$E_{\infty }(h,\tau )$	$\text{Space}_{\infty }$	$E_{\infty }(h,\tau )$	$\text{Space}_{\infty }$
		Our method		Our method		CNS [36]	
0.2	1/5	1.0859e−02		1.5316e−02		2.6917e−02	
	1/10	5.7595e−05	7.5587	7.7591e−05	7.6249	1.6360e−03	4.0403
	1/20	7.4037e−07	6.2816	1.0556e−06	6.1998	1.0096e−04	4.0183
0.4	1/5	1.0799e−02		1.5303e−02		2.6888e−02	
	1/10	5.7073e−05	7.5639	7.6880e−05	7.6370	1.6188e−03	4.0540
	1/20	7.3276e−07	6.2833	1.0440e−06	6.2024	1.0048e−04	4.0099
0.6	1/5	1.0697e−02		1.5252e−02		2.6750e−02	
	1/10	5.6275e−05	7.5705	7.5741e−05	7.6537	1.5965e−03	4.0666
	1/20	7.2262e−07	6.2831	1.0382e−06	6.1889	9.9534e−05	4.0036
(iii) Let $\tau =1/512$ be fixed. In Table 3, we compare $E^{\mathrm{Re}}_{\infty }(h,\tau )$ and $E^{\mathrm{Im}}_{\infty }(h,\tau )$ of our scheme (2.25)–(2.27) with those of the meshless collocation (MC) method [47] and the CNS method [36]. The numerical results indicate that our method gives the smallest errors in all the cases.

**Table 3:** (Example 6.1) Comparing $E^{\mathrm{Re}}_{\infty }(h,\tau )$ and $E^{\mathrm{Im}}_{\infty }(h,\tau )$ with other methods

*γ*	*h*	$E^{\text{Re}}_{\infty }(h,\tau )$	$E^{\text{Re}}_{\infty }(h,\tau )$	$E^{\text{Re}}_{\infty }(h,\tau )$	$E^{\text{Im}}_{\infty }(h,\tau )$	$E^{\text{Im}}_{\infty }(h,\tau )$	$E^{\text{Im}}_{\infty }(h,\tau )$
		Our method	MC [47]	CNS [36]	Our method	MC [47]	CNS [36]
0.1	1/9	1.2703e−04	7.0404e−02	1.2683e−03	1.2556e−04	7.6325e−02	2.4250e−03
	1/14	3.0566e−06	2.1873e−02	2.2077e−04	1.0443e−05	2.6090e−02	4.1972e−04
	1/19	4.1062e−07	1.0022e−02	6.5147e−05	1.4591e−06	1.2230e−02	1.2268e−04
	1/24	9.4755e−08	5.1958e−03	2.5400e−05	3.2571e−07	6.4207e−03	4.8343e−05
	1/29	6.5541e−08	2.8536e−03	1.1938e−05	1.0337e−07	3.5662e−03	2.2621e−05
0.3	1/9	1.2838e−04	7.0520e−02	1.3135e−03	1.2426e−04	3.5128e−02	2.3999e−03
	1/14	3.1059e−06	2.1979e−02	2.2990e−04	1.0361e−05	1.4733e−02	4.1516e−04
	1/19	4.0294e−07	1.0068e−02	6.7510e−05	1.5206e−06	7.1997e−03	1.2140e−04
	1/24	4.1570e−07	5.2146e−03	2.6167e−05	4.6692e−07	3.8478e−03	4.7855e−05
	1/29	4.3146e−07	2.8610e−03	1.2195e−05	2.9081e−07	2.1771e−03	2.2402e−05
(iv) In Table 4, we fix $(h,\tau )=(1/40,1/200)$ and compare $E^{\mathrm{Re}}_{\infty }(h,\tau )$ and $E^{\mathrm{Im}}_{\infty }(h,\tau )$ of our scheme (2.25)–(2.27) with those of the quadratic B-spline Galerkin (QBG) method [16] and the CNS method [36]. Once again, the numerical results show that our scheme outperforms these methods.

**Table 4:** (Example 6.1) Comparing $E^{\text{Re}}_{\infty }(h,\tau )$ and $E^{\text{Im}}_{\infty }(h,\tau )$ with other methods

*γ*	$E^{\text{Re}}_{\infty }(h,\tau )$	$E^{\text{Re}}_{\infty }(h,\tau )$	$E^{\text{Re}}_{\infty }(h,\tau )$	$E^{\text{Im}}_{\infty }(h,\tau )$	$E^{\text{Im}}_{\infty }(h,\tau )$	$E^{\text{Im}}_{\infty }(h,\tau )$
	Our method	QBG [16]	CNS [36]	Our method	QBG [16]	CNS [36]
0.10	3.1730e−07	4.6850e−04	3.1153e−06	1.5460e−07	7.7635e−04	6.2728e−06
0.30	2.1533e−06	4.9949e−04	3.3537e−06	1.1104e−06	3.2833e−04	6.5370e−06
0.70	3.9262e−05	6.6590e−04	3.9311e−05	3.2046e−05	1.0614e−03	3.2046e−05
0.90	1.9926e−04	8.4460e−04	1.9971e−04	1.4957e−04	1.3150e−03	1.4957e−04
(v) To visualize the efficiency of our scheme (2.25)–(2.27), we plot the real part (imaginary part) of the numerical solution and exact solution in Fig. 1 (Fig. 2) for $\gamma =0.1$ and $(h,\tau )=(1/16,1/200)$. From the figures, we observe that our method gives a good approximation of the exact solution.

### Example 6.2

Consider the time-fractional Schrödinger equation

6.8 $$ \textstyle\begin{cases} \displaystyle i\,{}^{C}_{0}{D}^{\gamma }_{t} u(x,t)+ \frac{\partial ^{2} u(x,t)}{\partial x^{2}}+ \big\vert u(x,t) \big\vert ^{2}u(x,t)=f(x,t), \quad (x,t)\in [0,1]\times [0,1], \\ u(x,0)=0, \quad x\in [0,1], \\ u(0,t)=it^{\gamma +1}, \qquad u(1,t)=it^{\gamma +1}, \quad t\in [0,1], \end{cases} $$

where $0<\gamma <1$ and

6.9 $$ \begin{aligned} f(x,t)&= t\Gamma (\gamma +2) \bigl[i\sin (2\pi x)-\cos (2\pi x) \bigr] \\ &\quad {}+ \bigl(t^{3 \gamma +3}-4\pi ^{2}t^{\gamma +1} \bigr) \bigl[\sin (2\pi x)+i\cos (2\pi x) \bigr]. \end{aligned} $$

The exact solution is $u(x,t)=t^{\gamma +1}[\sin (2\pi x)+i\cos (2\pi x)]$.

**Figure 4:**
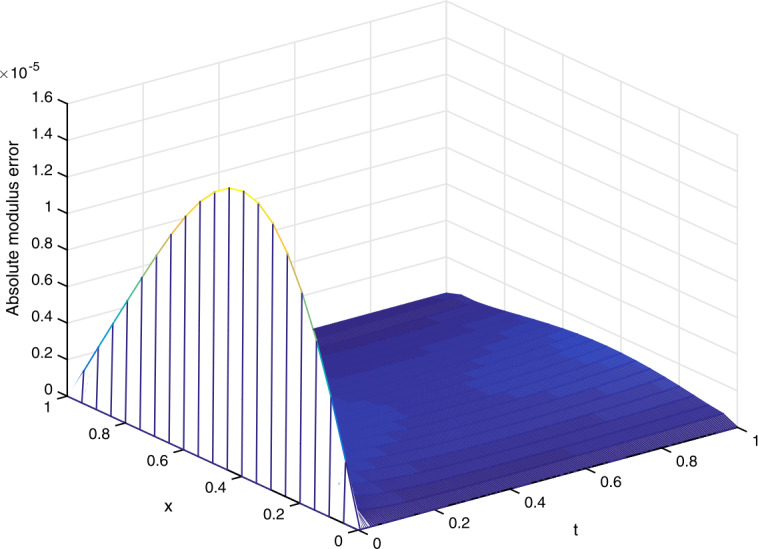
(Example 6.2) Absolute modulus error

Let $\alpha =1/20$ in the implementation of the scheme (2.25)–(2.27). In Table 5, we fix $\tau =1/5000$ and present the spatial convergence orders of our scheme and those of the CNS method [36]. In Table 6, we fix $\tau =1/512$ and compare the maximum real/imaginary part absolute errors of our numerical scheme with those of the CNS method [36]. Furthermore, in Fig. 4 we plot the absolute modulus error $|u_{j}^{n}-U_{j}^{n}|$ obtained from our scheme for $\gamma =0.3$ and $(h,\tau )=(1/20,1/200)$.

**Table 5:** (Example 6.2) $E_{2}(h,\tau )$, $E_{\infty }(h,\tau )$ and spatial convergence orders

*γ*	*h*	$E_{2}(h,\tau )$	Space_2_	$E_{\infty }(h,\tau )$	$\text{Space}_{\infty }$	$E_{\infty }(h,\tau )$	$\text{Space}_{\infty }$
		Our method		Our method		CNS [36]	
0.3	1/5	1.0859e−02		1.5317e−02		2.6918e−02	
	1/10	5.7585e−05	7.5590	7.7577e−05	7.6253	1.6327e−03	4.0432
	1/20	7.3933e−07	6.2833	1.0550e−06	6.2003	1.0095e−04	4.0155
0.5	1/5	1.0808e−02		1.5308e−02		2.6893e−02	
	1/10	5.7103e−05	7.5643	7.6944e−05	7.6363	1.6200e−03	4.0532
	1/20	7.3204e−07	6.2855	1.0429e−06	6.2051	1.0053e−04	4.0103
0.7	1/5	1.0698e−02		1.5253e−02		2.6736e−02	
	1/10	5.6084e−05	7.5755	7.5675e−05	7.6551	1.5956e−03	4.0666
	1/20	7.6999e−07	6.1866	1.2828e−06	5.8824	9.9461e−05	4.0038

**Table 6:** (Example 6.2) $E^{\text{Re}}_{\infty }(h,\tau )$ and $E^{\text{Im}}_{\infty }(h,\tau )$

*γ*	*h*	$E^{\text{Re}}_{\infty }(h,\tau )$	$E^{\text{Re}}_{\infty }(h,\tau )$	$E^{\text{Im}}_{\infty }(h,\tau )$	$E^{\text{Im}}_{\infty }(h,\tau )$
		Our method	CNS [36]	Our method	CNS [36]
0.1	1/9	1.2677e−04	1.2598e−03	1.2575e−04	2.4285e−03
	1/14	3.0465e−06	2.1903e−04	1.0459e−05	4.2040e−04
	1/19	6.4303e−07	6.4687e−05	1.4559e−06	1.2287e−04
	1/24	6.4494e−07	2.5240e−05	4.0624e−07	4.8414e−05
	1/29	6.4482e−07	1.1872e−05	4.0832e−07	2.2651e−05
0.3	1/9	1.2773e−04	1.2910e−03	1.2491e−04	2.4131e−03
	1/14	4.6524e−06	2.2543e−04	1.0386e−05	4.1757e−04
	1/19	4.6830e−06	6.6416e−05	5.1023e−06	1.2207e−04
	1/24	4.6805e−06	2.5865e−05	5.1264e−06	4.8095e−05
	1/29	4.6723e−06	1.2160e−05	5.1146e−06	2.2506e−05

From the numerical simulation and plot, once again we demonstrate that the theoretical spatial convergence order of our scheme is at least six (Theorem 5.1) and our scheme performs better than the CNS method.

In the next example, we shall investigate the effect of *α* on the actual maximum *L*2 norm error $E_{2}(h,\tau )$.

### Example 6.3

Consider the time-fractional Schrödinger equation

6.10 $$ \textstyle\begin{cases}\displaystyle i\,{}^{C}_{0}{D}^{\gamma }_{t} u(x,t)+ \frac{\partial ^{2} u(x,t)}{\partial x^{2}}+ \big\vert u(x,t) \big\vert ^{2}u(x,t)=f(x,t), \quad (x,t)\in [0,1]\times [0,1], \\ u(x,0)=0, \quad x\in [0,1], \\ \displaystyle u(0,t)=i\bigl(t^{\gamma +2}-t^{2}\bigr), \qquad u(1,t)=i\bigl(t^{\gamma +2}-t^{2}\bigr), \quad t \in [0,1], \end{cases} $$

where $0<\gamma <1$ and

6.11 $$ \begin{aligned} f(x,t)&= \biggl[\frac{\Gamma (\gamma +3)}{\Gamma (3)}t^{2}- \frac{\Gamma (3)}{\Gamma (3-\gamma )}t^{2-\gamma } \biggr] \bigl[i\sin (2 \pi x)-\cos (2\pi x) \bigr] \\ &\quad {} + \bigl[-4\pi ^{2} \bigl(t^{\gamma +2}-t^{2} \bigr)+ \bigl(t^{\gamma +2}-t^{2} \bigr)^{3} \bigr] \bigl[\sin (2\pi x)+i\cos (2\pi x) \bigr]. \end{aligned} $$

The exact solution is $u(x,t)=(t^{\gamma +2}-t^{2})[\sin (2\pi x)+i\cos (2\pi x)]$.

**Figure 5:**
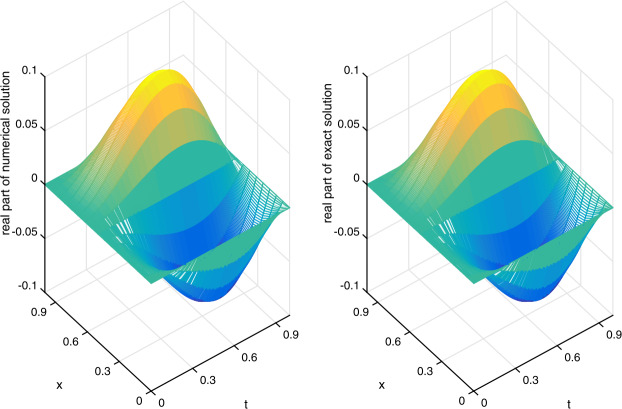
(Example 6.3) Real part of numerical solution and exact solution

**Figure 6:**
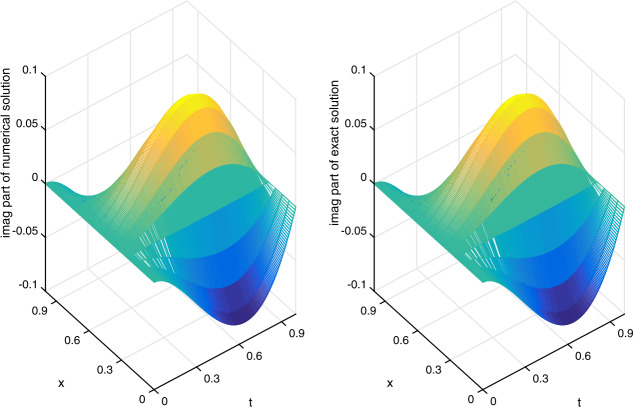
(Example 6.3) Imaginary part of numerical solution and exact solution

**Figure 7:**
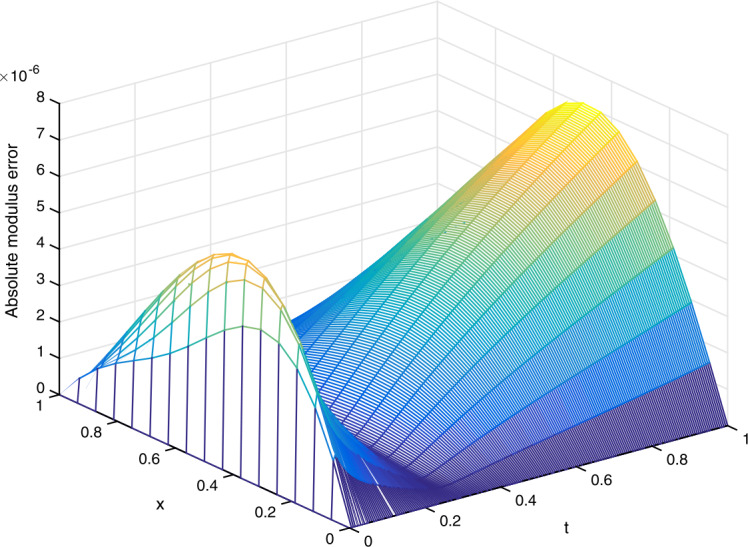
(Example 6.3) Absolute modulus error

First, we let $\alpha =1/20$ and $\tau =1/5000$ and present the spatial convergence orders of our scheme (2.25)–(2.27) and those of the CNS method [36] (Table 7). To visualize the efficiency of our method, in Figs. 5–7 we plot the real/imaginary parts of the numerical and exact solutions and the absolute modulus error for $\gamma =0.5$ and $(h,\tau )=(1/16,1/200)$. We observe that the theoretical spatial convergence of our method is indeed at least $O(h^{6})$ (Theorem 5.1) and our method gives a good approximation of the exact solution.

**Table 7:** (Example 6.3) $E_{2}(h,\tau )$, $E_{\infty }(h,\tau )$ and spatial convergence orders

*γ*	*h*	$E_{2}(h,\tau )$	Space_2_	$E_{\infty }(h,\tau )$	$\text{Space}_{\infty }$	$E_{\infty }(h,\tau )$	$\text{Space}_{\infty }$
		Our method		Our method		CNS [36]	
0.1	1/5	1.6057e−04		2.4239e−04		3.8406e−04	
	1/10	8.4508e−07	7.5699	1.0992e−06	7.7847	2.3556e−05	4.0272
	1/20	1.0644e−08	6.3110	1.5054e−08	6.1902	1.4549e−06	4.0171
0.3	1/5	4.6080e−04		6.9537e−04		1.1029e−03	
	1/10	2.4213e−06	7.5705	3.1527e−06	7.7850	6.7549e−05	4.0292
	1/20	3.0459e−08	6.3128	4.3067e−08	6.1939	4.1711e−06	4.0174
0.5	1/5	7.4202e−04		1.1157e−03		1.7736e−03	
	1/10	3.8806e−06	7.5790	5.0736e−06	7.7807	1.0865e−04	4.0289
	1/20	5.3287e−08	6.1864	7.6384e−08	6.0536	6.6960e−06	4.0202

**Figure 8:**
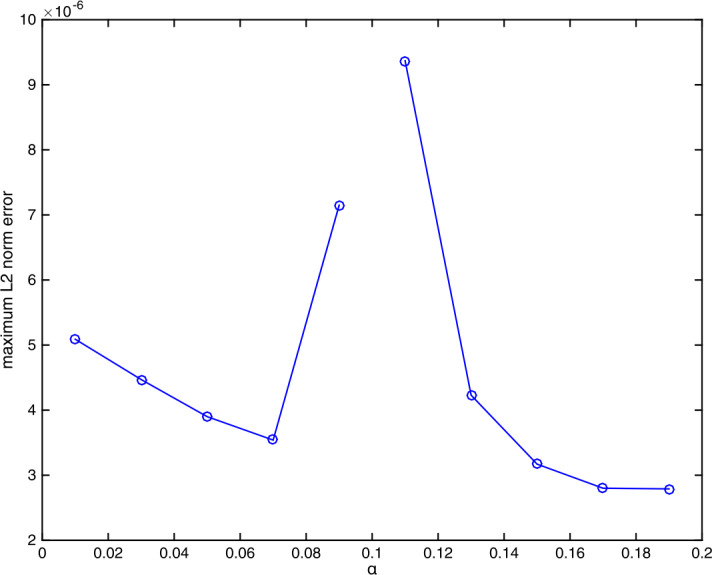
(Example 6.3) Influence of *α* on the error $E_{2}(h,\tau )$

Next, we shall investigate the influence of *α* on the error $E_{2}(h,\tau )$. Consider the case when $\gamma =0.5$ and $(h,\tau )=(1/10, 1/5000)$. We apply our scheme (2.25)–(2.27) with different values of $\alpha \in (0,\frac{1}{10} )\cup (\frac{1}{10}, \frac{37}{180} )$ and plot the error $E_{2}(h, \tau )$ against *α* in Fig. 8. It is observed that the exponent of the error remains the same $(10^{-6})$ for all $\alpha \in (0,\frac{1}{10} )\cup (\frac{1}{10}, \frac{37}{180} )$. Noting Table 7, we see that *regardless* of the chosen value of *α*, our method (error ∼10^−6^) is better than the CNS method (error ∼10^−4^) in this case.

## Conclusion

In this paper, we derive a numerical scheme to solve the time-fractional nonlinear Schrödinger equation (1.1) of fractional order $\gamma \in (0,1)$. Our tools include the quintic non-polynomial spline and *L*1 discretization. The unconditional stability, unique solvability and convergence are proved by the Fourier method. It is shown that our method can achieve sixth order convergence in space and $(2-\gamma )$th order convergence in time. Three numerical examples are presented to verify the theoretical results and to compare with other methods in the literature.
